# Paf15 expression correlates with rectal cancer prognosis, cell proliferation and radiation response

**DOI:** 10.18632/oncotarget.9606

**Published:** 2016-05-26

**Authors:** Rong Yan, Kun Zhu, Chengxue Dang, Ke Lan, Haonan Wang, Dawei Yuan, Wei Chen, Stephen J. Meltzer, Kang Li

**Affiliations:** ^1^ Department of Surgical Oncology, The First Affiliated Hospital, Xi'an Jiaotong University College of Medicine, Xi'an, Shaanxi, China; ^2^ Departments of Medicine (GI Division) and Oncology, Johns Hopkins School of Medicine and Sidney Kimmel Comprehensive Cancer Center, Baltimore, MD, USA

**Keywords:** Paf15, rectal cancer, prognostic factor, gamma radiation, cell cycle

## Abstract

Paf15, which participates in DNA repair, is overexpressed in numerous solid tumors. Blocking of Paf15 inhibits the growth of many types of cancer cells; while simultaneously enhancing cellular sensitivity to UV radiation. However, its expression and function in rectal cancer (RC) remain unknown. The current study was undertaken to assess the association of Paf15 expression with RC prognosis, as well as to explore the participation of Paf15 in the response of RC cells to irradiation. Increased Paf15 expression was observed in RC tissues and associated with pTNM stage and poor survival. *In vitro*, Paf15 induced increased RC cell proliferation while accelerating cell cycle progression, inhibiting cell death, and protecting against gamma radiation-induced DNA damage in RC cells. In conclusion, increased Paf15 expression is associated with increased RC proliferation, decreased patient survival, and a worse radiotherapeutic response.

## INTRODUCTION

Paf15 (also known as KIAA0101, NS5ATP9, OEACT-1, and L5), is a 12-kDa proliferating cell nuclear antigen (PCNA) - associated protein that is involved in cancer development and progression [1]. Paf15 is overexpressed in a variety of solid cancer tissues [2–10] and confers a growth advantage on cancer cells. Its functions also include the promotion of DNA repair, cell proliferation, cell cycle progression, and cell migration [1, 2, 11–13].

Rectal cancer (RC) is one of the most prevalent cancers in the world, and its mortality has continued to rise in the Asia-Pacific region [14]. To date, there have been several published studies measuring Paf15 expression in cancer patient tissues, such as lung, thyroid, hepatocellular, breast, adrenal, gastric and esophageal cancers. [2, 5, 7–10], but rectal cancer is not among these studies. One study evaluated mRNA levels of Paf15 in circulating plasma samples from colorectal cancer patients [15]. However, without matched data on protein and mRNA expression in tissues, peripheral blood mRNA levels may not reflect the disease conditions studied. The current study was performed to elucidate the clinical significance of Paf15 in RC patient tissue samples, specifically to assess the relationship between Paf15 expression, radiation response, and prognosis.

Recently, overexpression of Paf15 was shown to protect cells from UV radiation-induced cell death [16–18]. In certain conditions, UV-induced apoptosis is significantly decreased by Paf15's competition with p21 by binding PCNA [18, 19]. However, to our knowledge, the expression and function of Paf15 during clinical radiation therapy of patients has never been investigated.

Thus, rectal cancer cell lines were studied to clarify the role of Paf15 on the response to of gamma radiation in the current study. The reasons for selecting rectal cancer were: 1. Radiotherapy and neo-adjuvant radiotherapy for RC is routinely administered in the clinic, but radiotherapy resistance is commonly encountered; 2. We could find no published articles addressing the relationship between Paf15 and DNA repair or radiation-induced DNA damage in RC.

## RESULTS

### Paf15 expression in paired RC tissue samples

**Figure 1:**
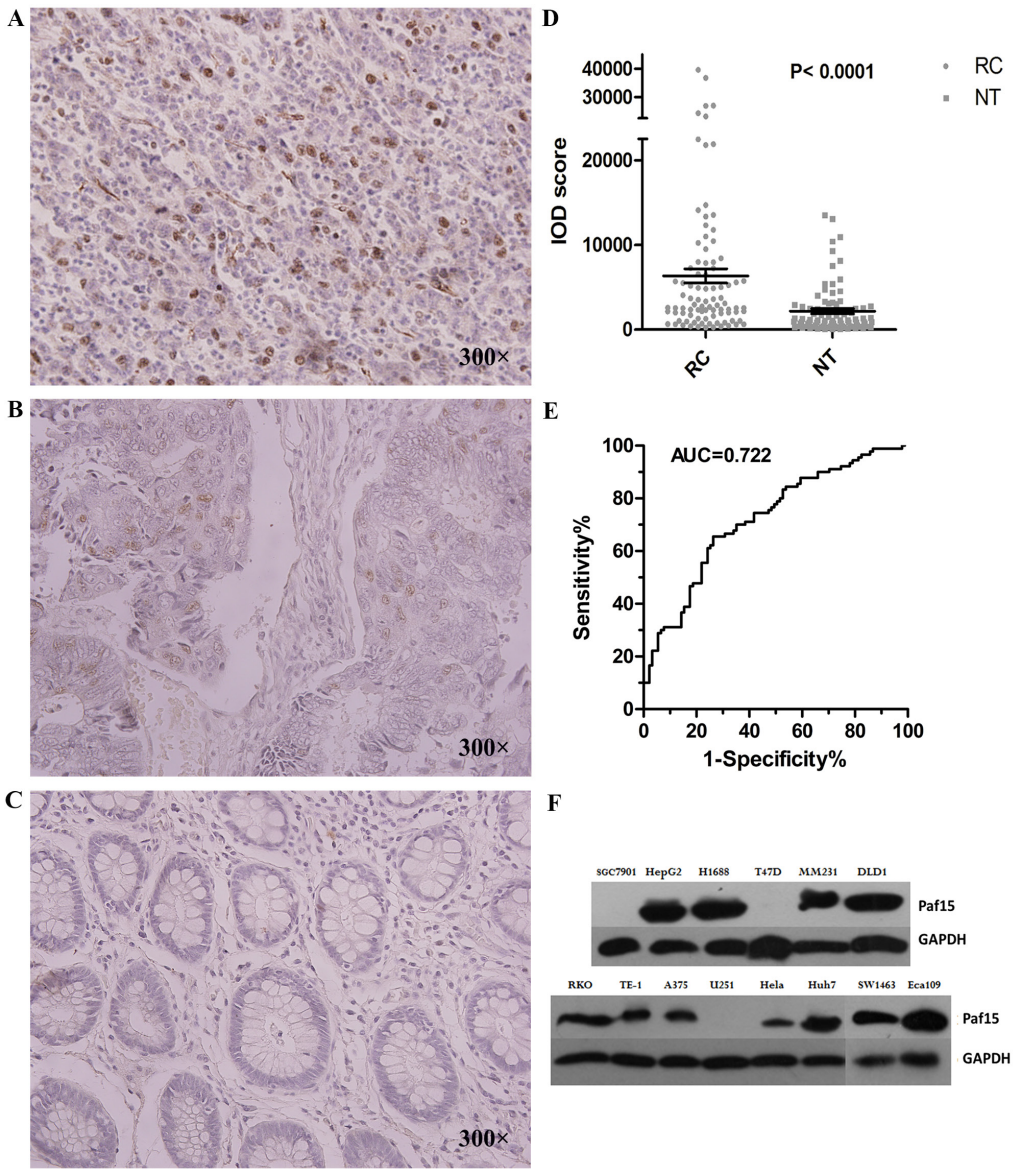
Expression of Paf15 in RC **A.** Strong positive image in RC. Staining is indicated by brown color in nuclei. **B.** Modest positive image: several cells showed less intense nuclear staining. **C.** Negative Paf15 expression. **D.** Paf15 expression levels in paired normal colorectal cancer and adjacent normal tissue (n = 105) as shown normalized by IOD score. **E.** ROC curve analysis for Paf15 expression. The area under the curve (AUC) was 0.722; **F.** Paf15 expression levels in multiple cancer cell lines.

The expression of Paf15 was assessed by Immunohistochemistry (IHC) on all 105 paired samples. Positive staining was seen by the naked eye in 53 (50.5%) of 105 RC samples and 21 (20.0%) of 105 matched non-cancer rectal tissues. As shown in Figure 1A–1C, Paf15 was expressed principally in tumor cell nuclei, while expression intensity and proportion varied among samples. Scattered expression was also observed in normal rectal mucosal cells and mucosa-associated lymphoid follicles (Supplementary Figure S1). Next, captured images were scored using Image Pro-Plus 14 software. IOD scores, representing density mean, area sum, and integrated optical density of positive staining (as described in Materials and Methods) were used for measurement. Results ranged from 19.8 to 39,578, with a median of 2,923.2. A statistically significant difference (P<0.0001; paired T-test) was found between RCs and paired normal tissues (Figure 1D). An ROC curve was also drawn (Figure 1E): AUC = 0.722 for the ability of Paf15 IHC expression to distinguish cancer from paired normal tissue. In all, 99 (94.29%) of 105 patients exhibited higher IOD scores in RC tissue than in paired normal tissue. Then, Paf15 Western blotting was performed in multiple cancer cell lines to check the specificity of the Paf15 antibody. As shown in Figure 1F, variable expression of Paf15 was observed in these cell lines.

### Paf15 expression *vs.* clinical parameters

We next explored whether Paf15 expression correlated with patient-associated clinical factors, including age, gender, histological grade, tumor size, lymph node invasion and TNM stage. Paf15 IHC expression levels were divided into two groups by median IOD score, as measured in both RCs and paired non-cancer tissues (Table 1). We did not find any significant correlation between clinical factors and Paf15 expression in paired non-cancer tissues. Therefore, the following results refer only to Paf15 expression in RC tissues.

**Table 1:** Associations between Paf15 IOD scores and clinicopathological features in RC IHC samples (n = 105)

Prognostic variables	No. of cases	*Paf15-H in RC¶(%)*	P-value (χ^2^/multi)	*Paf15-H in AT§(%)*	P-value (χ^2^/multi)
**Gender**			0.498/0.945		0.118/0.388
**male**	56	30 (53.57)		8(14.29)	
**female**	49	23 (46.94)		13(26.53)	
**Age**			0.494/0.170		0.435/0.978
**≥65**	52	28 (53.85)		12(23.08)	
**<65**	53	25 (47.17)		9(16.98)	
**Histological grade**			0.193/0.788		0.114/0.050
**G1-G2**	26	10(38.46)		8(30.77)	
**G3-G4**	79	42(53.16)		13(16.46)	
**Infiltration degree**			0.242/0.231		0.272/0.184
**T1-T2**	21	13(61.90)		6(28.57)	
**T3-T4**	84	40(47.62)		15(17.86)	
**Lymph node metastasis**			0.0001*/0.337		0.373/0.999
**+**	44	11(25.00)		7(15.91)	
**-**	61	42(68.85)		14(22.95)	
**pTNM Stage**			0.0001*/0.0001*		0.279/0.999
**I-II**	46	12(26.09)		14(23.73)	
**III-IV**	59	41(69.49)		7(15.22)	
**Tumor size**			0.944/0.004*		0.918/0.777
**<15cm^3^**	36	18(50.00)		7(19.44)	
**≥15cm^3^**	69	35(50.72)		14(20.29)	

* Correlation significant at the 0.01 level (two tailed); pTNM, pathological tumor/node/metastasis; multi, multivariate logistic regression analysis.

¶ Number of Paf15 high expression (IOD > median) cases and the percentage in RC tissues.

§ Number of Paf15 high expression (IOD > median) cases and the percentage in matched adjacent non-cancerous rectal tissues.

As demonstrated in Table 1, by χ^2^ testing, elevated Paf15 expression significantly correlated with pTNM stage (P < 0.001) and lymph node metastasis (P < 0.001). By multivariate logistic regression analysis, Paf15 expression correlated with pTNM stage (P < 0.001) and tumor size (P < 0.01). Age, gender, histological grade, tumor size and infiltration degree did not show any significant correlation with Paf15 expression. Finally, logistic regression identified pTNM stage as an independent predictive factor in cases within the Paf15-High group.

### Paf15 expression *vs.* survival

**Figure 2:**
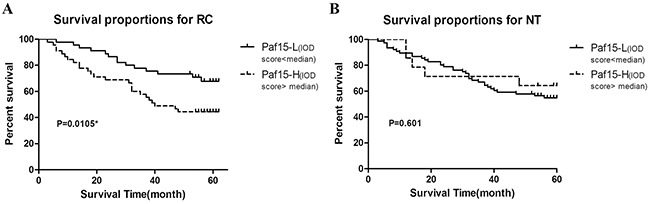
5-year survival in RC patients according to Paf15 expression level **A.** 5-year survival according to their tumor tissue (RC) Paf15 expression level. The survival rate was lower in patients with high than with low Paf15 IOD scores than patients with low Paf15 IOD scores (P = 0.0105). The 5-year survival rate for patients with Paf15-Low was 67.8%, and for patients with Paf15-High was 44.4%. **B.** 5-year survival according to their normal tissue (NT) Paf15 expression level. There was no statistically significant difference between 2 groups (P = 0.601).

In parallel, we assessed Paf15 expression *vs.* survival at 60 months follow-up. 39 (37.7%) of 105 patients died, while 15 patients lost to follow-up. Kaplan–Meier analyses were performed for Paf15 expression in RCs and paired non-cancer tissues (Figure 2). Results showed that overall survival was significantly longer when Paf15 expression level was high in RCs (P<0.05; log-rank test), but not in adjacent non-cancer tissues (P = 0.601; log-rank test). Thus, all further analyses focused on Paf15 IHC expression in cancer tissues.

**Figure 3:**
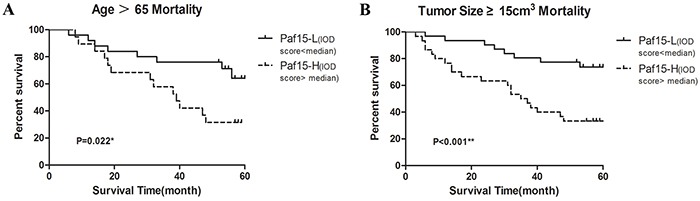
Subgroup analyzes of 5-year survival **A.** Subgroup analysis of patients older than 65. There was a statistically significant difference (P=0.022). The 5-year survival rate was 64.1% for patients with Paf15-Low and 31.6% for patients with Paf15-High. **B.** Subgroup analysis of tumor size ≥15cm^3^. There was a statistically significant (P<0.001). The 5-year survival rate was 73.7% for patients with Paf15-Low and 33.3% for patients with Paf15-High.

Next, several subset analyses were performed to assess the correlation between prognosis and Paf15 expression IOD score (Paf15-High *vs.* Paf15-Low) (Figure 3). Among patients older than 65, Paf15-High was significantly associated with shorter survival than Paf15-Low (P = 0.022, log-rank test). Among those whose tumor size was larger than 15 cm^3^, Paf15-High was also significantly associated with shorter survival (P < 0.001, log-rank test). However, there were no differential outcomes in any other subset analyses (Supplementary Figure S2A–S2H). As shown in Figure 3, age and tumor size were significantly associated with survival in Kaplan–Meier curves, but the remaining subgroups did not show a significant effect on survival.

Finally, backward stepwise Cox regression analysis was performed on the above data (Table 2). Survival was influenced only by Paf15 expression (P < 0.05), suggesting that Paf15 expression is an independent risk factor for RC survival. However, risk ratio failed to achieve a satisfactory value to claim correlation strength with survival time.

**Table 2:** Cox proportional hazards regression model of prognostic variables for overall survival

Prognostic variable	P-value	Risk ratio(95% CI)
**Paf15**	0.028*	0.479(0.248-0.923)
**Gender**	0.734	1.133(0.551-2.331)
**Age**	0.237	0.677(0.355-1.292)
**Histological grade**	0.197	0.501(0.175-1.433)
**Tumor size**	0.461	0.781(0.375-1.629)
**pTNM Stage**	0.327	0.695(0.336-1.438)
**Infiltration degree**	0.092	3.409(0.819-14.196)
**Lymph node metastasis**	0.586	0.563(0.071-4.457)

* Correlation significant at the 0.05 level (two tailed). CI: confidence interval.

### Paf15 promotes proliferation of RC cells in vitro

**Figure 4:**
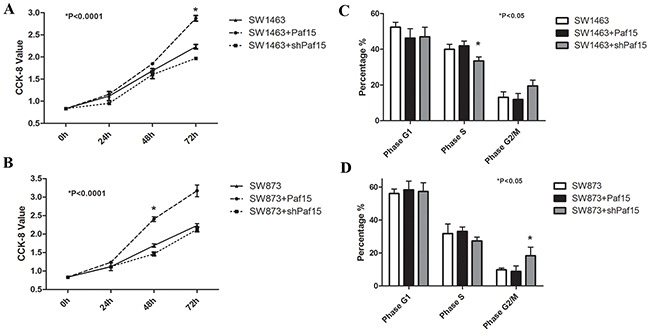
Paf15 expression influences on RC cell viability & cell cycle Cell viability was detected at 0, 24, 48 and 72 hours after transfection using a CCK-8 assay. There were statistically significant differences (P<0.001) in SW1463 at 72 hours **A.** and in SW837 at 48 hours **B.** among the 3 groups. Cell cycles were tested at 48 hours after transfection; SW1463 showed a statistically significant difference (P<0.05) in S phase among the 3 groups **C.** while SW837 showed a statistically significant difference (P<0.05) in G2/M phase among the 3 groups **D.**

Two RC cell lines (SW1463 and SW873) were studied. We divided each RC cell line into 3 groups in triplicate and separately transfected the Paf15+, shPaf15, and empty plasmids into them for comparison. Cell count and CCK-8 assay were used to assess cell viability. 48 h after transfection, a recognizable difference in Paf15 expression was observed by Western blotting (Supplementary Figure S4). Cell counts showed that the Paf15 up-regulated group exhibited reduced cell numbers by 14.7% in SW-1463 and by 17.2% in SW873 *vs.* the Paf15 down-regulated group. CCK-8 assays were performed at 24, 48 and 72 h after transfection. As shown in Figure 4A & 4B, a significant reduction in cell viability was seen in the shPaf15 group *vs.* the Paf15+ group (two-way ANOVA), suggesting that forced Paf15 overexpression confers a growth advantage on RC cells.

### Paf15 alters cell cycle distribution in RC cells *in vitro*

To further explore the function of Paf15 in RC cells, stably transfected RC cells were subjected to flow cytometry to detect early apoptosis and cell cycle distribution. As shown in Figure 4C & 4D, Paf15 did not change G1 phase among groups. Suppression of Paf15 increased the proportion of cells in G2/M phase and suppressed the proportion in S phase; however, this result was not consistent between the two cell lines.

Next, to determine whether decreasing Paf15 caused or was merely an effect of cell cycle change, we blocked the cell cycle in wild-type control RC cells using aphidicolin (1 μg/ml) or nocodazole (50 ng/ml) for 20 h to arrest cells in S or G2/M phases, respectively [20]. Paf15 expression levels were then measured by Western blotting (Supplementary Figure S5). After we artificially stalled RC cells at the S or G2/M phase, we failed to find a clear difference in Paf15 expression between them and unarrested control cells, suggesting that altered Paf15 expression was a cause of the cell cycle changes, rather than a consequence of them. Moreover, when we focused on early apoptosis results, we discovered relatively diminished apoptosis (by PI) in the Paf15+ group (P < 0.05), suggesting that Paf15 suppresses apoptosis or necrosis in RC cells.

### Paf15 expression after gamma irradiation & effect on DNA damage

To investigate whether Paf15, as in previous UV studies, is involved in gamma irradiation-induced DNA damage repair, we first established a model of DNA damage by gamma irradiation, as described in Materials and Methods. The dosage chosen for this experiment was 8 Gy.

**Figure 5:**
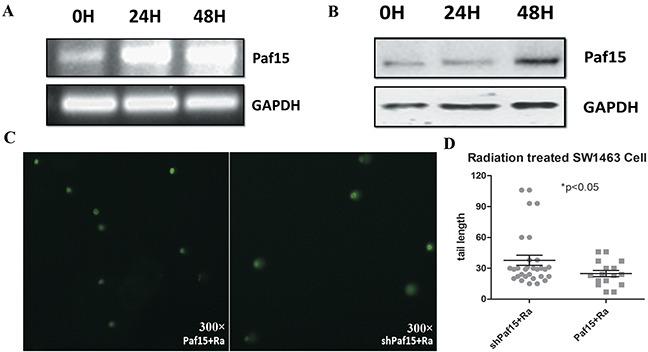
Paf15 expression after radiation and its effect on DNA damage **A.** Paf15 expression at 0, 24 and 48 hours after radiation was measured using RT-PCR (A) and Western blot **B.** with GAPDH expression as a control. **C.** DNA damage at 48 hours after radiation was compared for different Paf15 expression levels by comet assay. **D.** DNA damage measured by comet tail length of each measured cell. Increased Paf15 expression resulted in a reduction of DNA damage after radiation (P<0.05).

48 h after irradiation, a recognizable increase in Paf15 expression was observed by RT-PCR (normalized score: 0.49 for 0H; 0.93 for 24H; and 1.17 for 48H) & Western blotting (normalized score: 0.39 for 0H; 0.36 for 24H; and 1.05 for 48H) in wild-type RC cells (Figure 5A & 5B). Increased expression occurred more rapidly at the RNA level than at the protein level. This discrepant result may have been due to the ubiquitin-consuming effect on Paf15 during error-free repair after DNA damage [11]. The more rapid response of mRNA expression/degradation compared to protein could have accounted for this observation, too. In addition, we found that changes in cell morphology due to aging were apparent, as cytoplasmic granules and particles were increased [21].

In cells with different Paf15 expression levels, nucleic acid injury status also presented differently in comet assays (Figure 5C). Comet tail length, representing the degree of DNA damage, was significantly shorter in Paf15-up-regulated cells (Figure 5D). This difference could have reflected a reparative effect of Paf15 during radiotherapy-induced cell injury.

### Paf15 in post-irradiation cell cycle & cell proliferation

**Figure 6:**
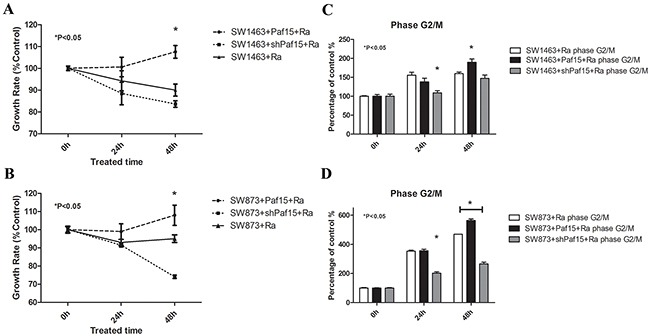
Effects of Paf15 expression on cell viability & cell cycle after radiation RC cells with varying Paf15 expression levels were treated with gamma irradiation or plated without radiation as a control, then subjected to CCK-8 assay & flow cytometry at 0, 24 and 48 hours. **A. & B.** Growth rate, expressed as CCK-8 value percentages of unirradiated controls, showed a significant difference in cell proliferation at 48 h after radiation (P<0.05). **C.** Decreased Paf15 expression alleviated radiation-induced G2/M phase arrest in SW1463 at 24 hours after radiation (P<0.05) and **D.** in SW873 at both 24 and 48 hours after radiation (P<0.05). Vertical axis represents G2/M phase cell percentages of unirradiated controls.

Cell cycle and proliferation were tested before radiation and 24 h or 48 h after radiation, respectively, by CCK-8 assay (Supplementary Figures S6A-S6F) and by flow cytometry (Supplementary Figures S7A–S7F). When expressed as percentages of controls, results showed a significant difference in cell proliferation at 48 h after radiation (Figure 6A & 6B), suggesting that Paf15 promotes RC cell proliferation after radiation.

In cell cycle tests, the percentage of cells in G2/M phase increased steadily with time (Supplementary Figures S7E & S7F), implying that G2/M phase arrest was induced by gamma irradiation [22]. Moreover, when expressed as a percentage of un-irradiated cells' G2/M phase, G2/M arrest was lower in the Paf15 down-regulated group, with a statistically significant difference at both time points in SW873 cells but only at 24 h in SW1463 cells (Figure 6C & 6D). Thus, G2/M phase proportion after radiation was diminished by suppression of Paf15 expression. In contrast, G1 phase and S phase did not change significantly over time post-irradiation (Supplementary Figure S8).

## DISCUSSION

In this study, we found that Paf15 was highly expressed in RC samples and detectable at lower levels in non-cancer rectal samples. Simpson *et al*. [18] demonstrated that Paf15 localizes to high-proliferation areas, suggesting that Paf15 overexpression facilitates cell proliferation. In the current study, our finding of detectable Paf15 expression in non-cancer tissues may reflect a growth advantage conferred by this gene. In agreement with our finding, previous research based on breast cancer tissue microarrays found a correlation between Paf15 overexpression and positive Ki67 staining [23] and emphasized its relationship to increased disease severity.

Our *in vitro* experiments showed a significant positive correlation between Paf15 expression and proliferation in RC cells. Cell cycle and apoptosis were also inhibited after increase Paf15 expression, indicating that Paf15 is involved in cell cycle progression. As previous studies have shown, Paf15 shares a PCNA-binding motif with other relevant cell cycle-regulatory PCNA-binding proteins, including p21, p57, and p33ING1b [18, 24–26]. The formation of p33ING1b and Paf15 complexes is thought to compete with p21^WAF1^, leading to ubiquitin-dependent p21^WAF1^ degradation and optimal DNA repair [17]. However, previous studies have expressed conflicting views regarding Paf15's distribution and influence on the cell cycle. One study showed that Paf15 protein is most abundant during S and G2 phases [23], mainly acting in the DNA damage response. Another study reported that inhibition of Paf15 reduces p21 protein binding to PCNA and decreases the number of cells in S phase, inducing arrest in G1 [7]. Still another study found that transfection of Paf15 cDNA inhibited HCC cell growth *in vitro* and arrested cells at the G1-S phase transition [27]. More thorough studies of Paf15-related molecular mechanisms are therefore needed.

In our experiments, we found that Paf15 inhibited DNA damage caused by gamma irradiation. In agreement with our results, Simpson *et al.* [18] reported that UV irradiation increases Paf15 expression and Paf15–PCNA complex formation, while acting on the DNA damage response. However, the principles of cell injury caused by gamma radiation *vs.* by UV are not identical. Radiation damages nuclear DNA, the cell membrane, and cytosolic proteins. Radiation-induced DNA damage mainly occurs as strand breaks, which cause cell division to be blocked, resulting in cell division failure or cell damage [28]; In contrast, 250nm – 260nm-wavelength UV destroys chromosomal DNA, which is called actinism. This reaction contains a series of processes including compounding, decomposition, ionization, and redox [29]. Thus, our results provide evidence that Paf15 could reduce more than one type of DNA damage. Moreover, expression of Paf15 in wild-type RC cells was stimulated by gamma irradiation in the current study. A marked increase impact of Paf15 on cell proliferation was seen after irradiation of RC cells in our study, indicating that inhibition of Paf15 enhances cellular sensitivity to gamma irradiation in RC cells.

Specifically, when DNA damage is induced by irradiation, a self-repairing process is launched in cells [30, 31]. The underlying mechanism of this reparative process is G1, S, or G2/M cell cycle arrest, which ensures that damaged cells have enough time to conduct self-repair, and thereby to generate resistance. This phenomenon is deemed beneficial in normal cells. However, in malignant cells, it has the potential to restrain signal-induced cell apoptosis and may enhance radiation resistance [30]. By down-regulating Paf15 expression, we may ultimately develop novel therapies in radiation-resistant tumors.

In conclusion, our results support the hypothesis that Paf15 is a key contributor to cancer cell proliferation and cell cycle progression. Paf15 expression in resected RC patient specimens may be a useful clinical prognostic index. *In vitro* results suggest that Paf15 could be mobilized to protect against gamma radiation-induced cell damage in RC. These experimental results suggest a future basis for deciding whether to administer radiotherapy in RC patients.

## MATERIALS AND METHODS

### Tissue samples and tissue microarray

Expression of Paf15 was assessed by immunohistochemistry in 105 paired human primary RC and normal rectal tissues (both in tissue microarrays and in surgically obtained samples). Samples in the tissue microarray were harvested at the Shanghai Oriental Hepatic Surgery Hospital between December 2008 and December 2009, while the remainders of samples were obtained during surgery on RC patients performed at the First Affiliated Hospital of Xi'an Jiaotong University from January 2012 to January 2013. All tumor samples were rectal adenocarcinoma and were histologically double-reviewed by pathologists for confirmation. All patients provided written informed consent. Tumors were staged according to the pathological tumor/node/metastasis (pTNM) classification (7th edition) [32]. Clinical parameters including age, gender, pathological type, pathological grading, infiltration degree, and tumor size were recorded at surgery. Patients were followed up regularly for more than 60 months or until death to analyze 5-year survival rate and recurrence. Cases treated pre-operatively with radiotherapy, and/or chemotherapy were excluded.

### Immunohistochemistry

Tissue specimens were embedded in paraffin and cut into serial 4-mm sections. Slides were immersed in 1 mmol EDTA (pH 9.0) and boiled for 15 min in a microwave oven. Endogenous peroxidase activity was blocked by incubation in 0.3% H_2_O_2_ in methanol for 15 min. Specimens were incubated with a primary antibody specific to Paf15 (AT2611a, 1:500 dilution; Abgent, San Diego, USA) at 4°C overnight. Then they were incubated with a secondary antibody (Goat Anti-Mouse IgG, 1:500 dilution; Pierce Biotechnology, Inc., Rockford, IL, USA) for 60 min at room temperature in a moist chamber. Slides were stained using diaminobenzidine tetrahydrochloride (DAB) and counterstained with hematoxylin. Next, they were dehydrated and slip-covered. Images of stained sections were obtained using an optical microscope (BX51; Olympus, Tokyo, Japan) equipped with a digital camera (PD71; Olympus). Paf15 was scored using Image Pro-Plus 6.0 [3 parameters: density mean, area sum, and integrated optical density (IOD)] [33], and results were confirmed by visual assessment. According to these IOD scores, Paf15 expression was divided into two groups: Paf15-Low (IOD score < median score); and Paf15-High (IOD score ≥ median score). All slides were independently assessed by two investigators (Dr. Kun Zhu and Rong Yan) to eliminate the scoring error. An agreement was reached after careful discussion.

### Statistical analysis

Paf15 immunoreactivity was assessed for association with clinical variables using the χ^2^ test. Next, multivariate stepwise logistic regression analysis was performed to identify independent variables that correlated with Paf15 expression level. The Kaplan–Meier method was adopted to generate survival curves, and prognostic variables were analyzed using the Cox proportional hazards regression model based on overall survival status. All statistical analyzes were conducted using SPSS 16.0 software (SPSS Chicago, IL). A value of P<0.05 was considered statistically significant.

### RNA preparation and q-PCR

Total RNA was extracted using Trizol (Invitrogen, Carlsbad, CA, USA) and reverse-transcribed using a First-strand cDNA Synthesis Kit (Fermentas, Burlington, ON, Canada). RNA quantity and quality were assessed by NanoDrop (NanoDrop Technologies, Inc., Thermo Fischer). SYBR Premix Ex Taq (TaKaRa, Ohtsu, Shiga, Japan) was used for qPCR analyzes. Experiments were undertaken with the synthesized Paf15 primers: 5′-TCCTGAAGAGGCAGGAAGCAGT-3′ and 5′- TTGT GTGATCAGGTTGCAAAGGA-3′; or with GAPDH- specific primers as an internal control: 5′-CGGAGT CAACGGATTTGGTCGTAT-3′ and 5′-AGCCTTCTC CATGGTGGTGAAGAC-3′. PCR reactions were optimized to ensure product intensity within the logarithmic phase of amplification. Final reactions were performed in a volume of 25 μL containing 1 μg of cDNA. Cycle parameters were denaturation for 15 s at 95°C, annealing for 20 s at 62°C, and extension for 20 s at 72°C for 40 cycles for Paf15; and denaturation for 15 s at 95°C, annealing for 20 s at 58°C, and extension for 20 s at 72°C for 35 cycles for GAPDH. Relative Paf15 expression levels were scored using Image J software, and results were confirmed by visual assessment.

### Protein preparation and western blotting

Tissues or cell lines were lysed in modified RIPA buffer (50 mM Tris,150 mM NaCl,1% Triton X-100, 1% sodium deoxycholate, and 0.1% SDS) containing 25 mg/ml leupeptin, 1 mM sodium orthovanadate, 2 mM EDTA, and 1 mM PMSF. Protein concentration was determined using a BCA kit (Pierce Biotechnology, Inc., Rockford, IL, USA). Protein samples (50 μg) were separated on 15% SDS-PAGE gels and transferred to polyvinylidene difluoride membranes (Sigma, St Louis, MO, USA). Membranes were incubated with a primary antibody specific to Paf15 (AT2611a, 1:500 dilution; Abgent, San Diego, USA) or GAPDH (1:1000; Santa Cruz Biotechnology, Inc., Santa Cruz, CA, USA) at 4°C overnight, then incubated with secondary antibody (Goat Anti-Mouse IgG, 1:500 dilution; Pierce Biotechnology, Inc.) for 1 h. Reactive proteins were detected using ECL reagents (Pierce Biotechnology, Inc.) and the G: BOX Bio Imaging System (Syngene, Cambridge, UK). Paf15 expression levels were determined by normalizing the signal intensity of Paf15 to that of GAPDH using Image J software. And results were confirmed by visual assessment.

### Cell culture and gene transfection

Human RC cell lines SW-1463 and SW-873 were obtained from the Type Culture Collection of the Chinese Academy of Sciences (Shanghai, China). Cells were grown in DMEM (High Glucose) medium supplemented with 10% heat-inactivated fetal bovine serum, 100 U/ml penicillin, and 100 mg/ml streptomycin at 37°C in a 5% CO_2_ incubator.

To induce varying expression levels of Paf15, full-length Paf15 cDNA was cloned into the expression vector p-EGFP as a Paf15 expression plasmid; and a pSIREN-Shuttle vector expressing a short hairpin RNA against Paf15 (5′-GCAACCTGATCACACAAATGA-3′) was also cloned into p-EGFP as a shPaf15 plasmid. Empty p-EGFP plasmid was used as a control. Plasmids carrying Paf15 cDNA or shRNA were separately transfected into the RC cell lines SW-1463 and SW-873 using Lipofectamine 2000 Reagent (Invitrogen, Carlsbad, CA, USA) according to the manufacturer's instructions. For stable transfection screening, cells were serially diluted and seeded onto 24-well plates containing 500 μl of culture medium with G418. The lowest concentration of G418 that caused massive cell death was determined. By maintaining selection pressure by keeping G418 in growth medium for 3-4 weeks, cells remained viable were determined to have retained expression plasmids, which stably integrate into the genome of host cells.

### Cell viability assessment

Cells containing Paf15 high expression or shRNA expression plasmids were assessed by the CCK-8 assay (Sigma-Aldrich, USA) for viability. Cells with empty plasmids served as controls. Experiments were performed according to the manufacturer's instructions in 96-well format in triplicate. One day before treatment, cells were seeded onto 96-well plates at a density of 3000 cells per well. Then, viability testing was implemented before and after 24, 48 or 72 h of treatment, respectively, for comparison. 20 μl of CCK-8 (10% in culture medium) were added to cells at each time point. Cells were then incubated for 2 h at 37°C. After agitation for 10 min on a shaker, absorbance at 562 nm was read using a scanning microtiter apparatus (Perkin Elmer, Waltham, MA, USA).

### Flow cytometric analyses

Cells were first treated with DNase-free RNase (100 mg/ml) for 20 min at 37°C, then stained with propidium iodide at a concentration of 50 mg/ml, then ethanol-fixed overnight at 4°C. Afterward, cells were suspended in 1x PBS to a concentration of 10^6^ cells/ml. Flow cytometric analyzes were performed on a Becton Dickinson FAC Scan (BD Biosciences, Franklin Lakes, NJ). Data files were generated for 15,000 events (cells) using CellQuest software. The fraction of the total cell population present in the G1, S, and G2/M cell cycle phases was obtained using Mod Fit LT software.

### Gamma radiation and establishment of dna damage model

According to published data, the optimal dosage of gamma radiation varies according to cell type and cancer type [34]. To discover the appropriate dose for this study, equal numbers of stably transfected cells were cultured in individual culture dishes. After reaching 70% confluence, cells were treated with 2 Gy, 4 Gy, 8 Gy, or 12 Gy, respectively. 4MV of energy, 100 cm of source-skin distance and 300 cGy/min of dose rate were routinely used to mimic clinical radiotherapy. Viability was analyzed by CCK-8 staining after 24 hours of incubation. The dosage that induced the biggest change in apoptosis was selected as the experimental dosage for subsequent studies.

Next, cells were passaged onto 6-well plates and divided into six groups after cell counting: (1) control group (C); (2) radiation-treated control group (C + Ra); (3) Paf15 up-regulated group (Paf15); (4) Paf15 up-regulated and radiation-treated group (Paf15 + Ra); (5) shPaf15 plasmid-transfected group (shPaf15); (6) shPaf15 plasmid-transfected and radiation-treated group (shPaf15 + Ra). Next, cells were starved for 72h in 0.5% fetal bovine serum DMEM medium to achieve cell cycle synchronization. Afterward, culture fluid was replaced with 10% bovine serum DMEM medium and cultured at 37°C for 1h. Finally, radiation interventions were implemented. Cell proliferation, cell cycle, and DNA damage were measured before and after varying times of damage repair (at 24 h and 48 h).

### Comet assay

Single-Cell Gel Electrophoresis (SCGE), also known as comet assay, was chosen to measure DNA damage in individual eukaryotic cells. The principle of the comet assay is that integrated DNA maintains a well-organized structure in the nucleus, but becomes disrupted when the cell is damaged [35]. This method detects both single- and double-strand DNA breaks, making it a useful technique for predicting the *in vivo* DNA response to gamma-irradiation.

Cells were detached by trypsinization (Trypsin-0.25% EDTA, 25200-072; Invitrogen) 48 h after irradiation. Alkaline lysis of cells, agarose gel slide preparation, and electrophoretic procedures were performed according to modified online procedures [36]. After electrophoresis, slides were observed under a fluorescence microscope. Analyzes of results were based on the percentage of DNA in the comet “head” (amount of genetic material distributed in the nucleus) and in “tail” (amount of genetic material distributed in the fragmented pieces), and tail length. Data were analyzed using Casplab Software version 1.2.3b.

## SUPPLEMENTARY FIGURES


