# Supplementary material for: Paf15 expression correlates with rectal cancer prognosis, cell proliferation and radiation response
Source: Oncotarget. 2016 May 26;7(25):38750–61. doi: 10.18632/oncotarget.9606 (PMC5122426; doi:10.18632/oncotarget.9606)
Supplement: Supplementary file 1 [file oncotarget-07-38750-s001.pdf]

Paf15 expression correlates with rectal cancer prognosis, cell proliferation and radiation response

SUPPLEMENTARY FIGURES

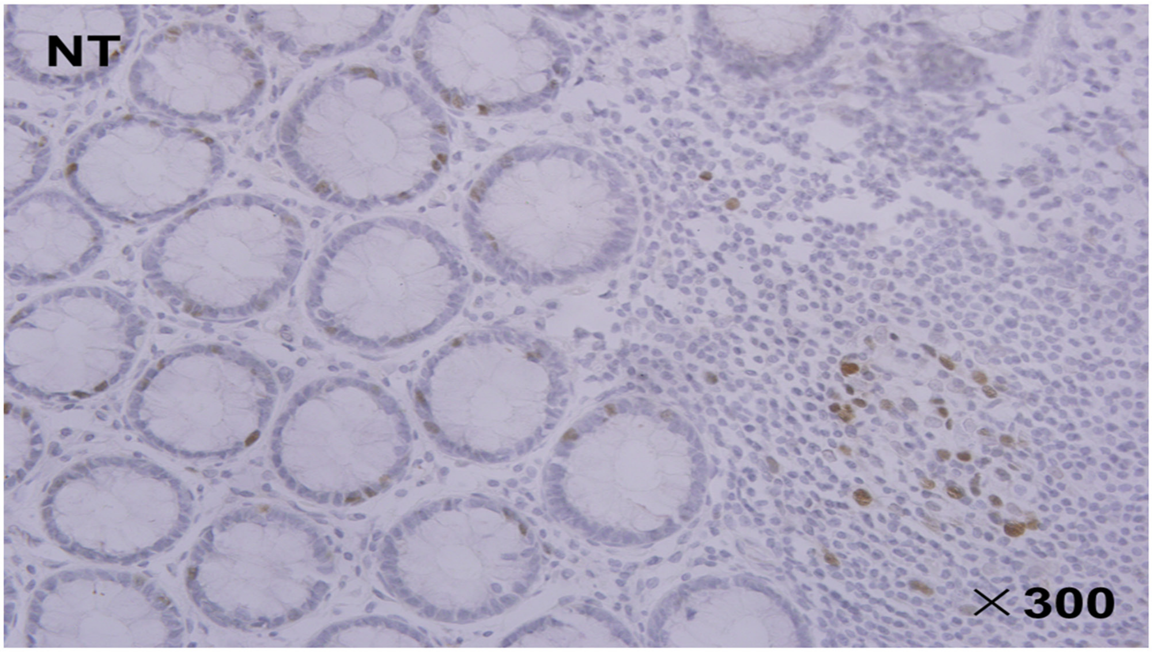

Supplementary Figure S1: Paf15 expression pattern in normal rectal tissue. Positive staining was found in normal rectal mucosal cells and mucosa-associated lymphoid follicles.

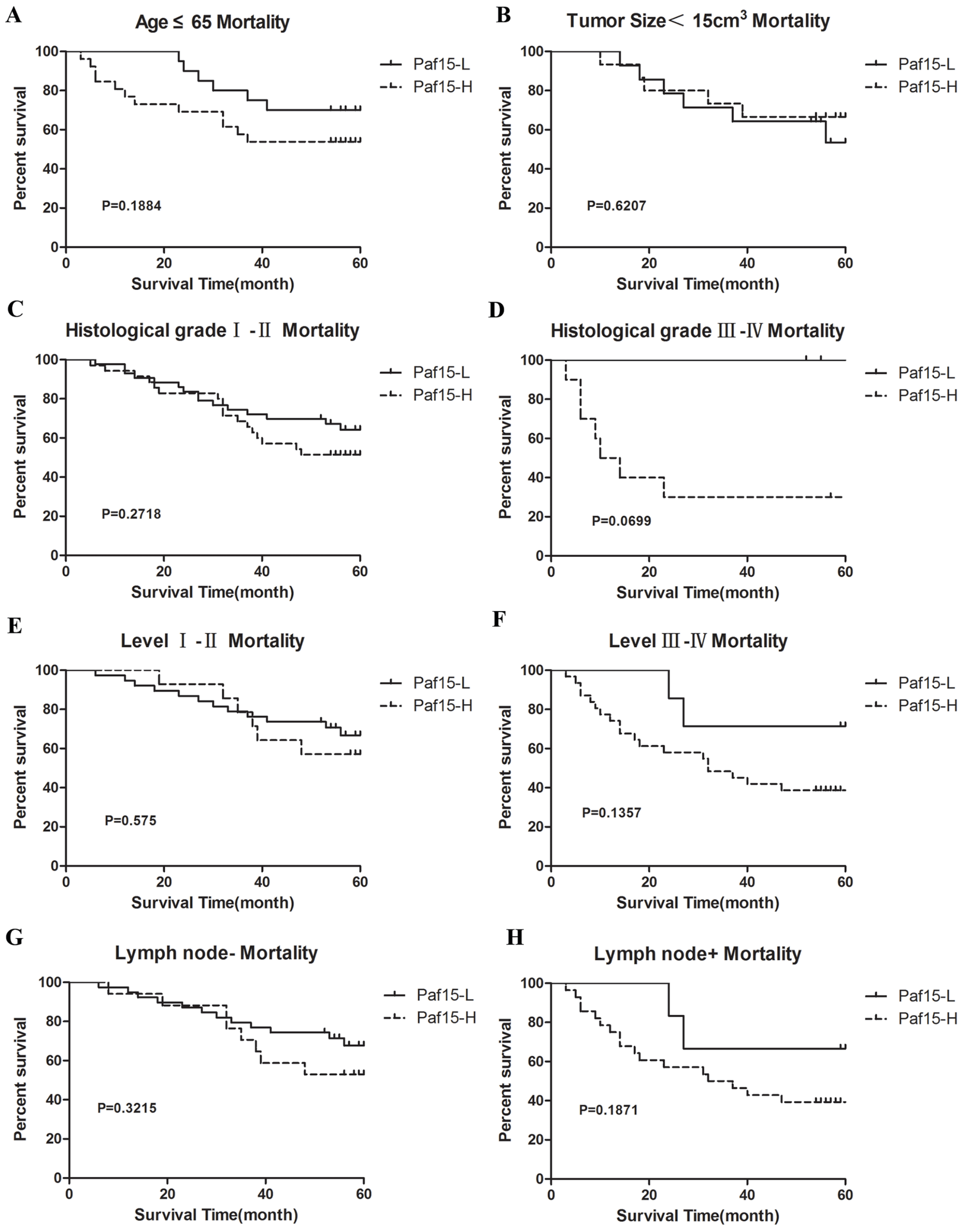

Supplementary Figure S2: Subgroup analyzes of 5-year survival. A. Subgroup analysis of patients younger than 65. **B.**Subgroup analysis of tumor size ≤15cm3. **C.** & **D.** Subgroup analyses of histological grade. **E.** & **F.** Subgroup analyses of pTNM stage. **G.** & **H.** Subgroup analyses of lymph node status. There were no statistically significant differences in any of these comparisons.

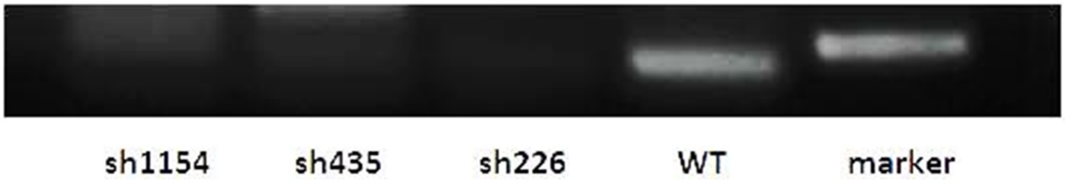

Supplementary Figure S3: Paf15 knockdown by different shPaf15 plasmids. Three different candidate shPaf15 plasmids were tested, and Paf15 expression was measured by RT-PCR. The plasmid chosen for this experiment was sh226.

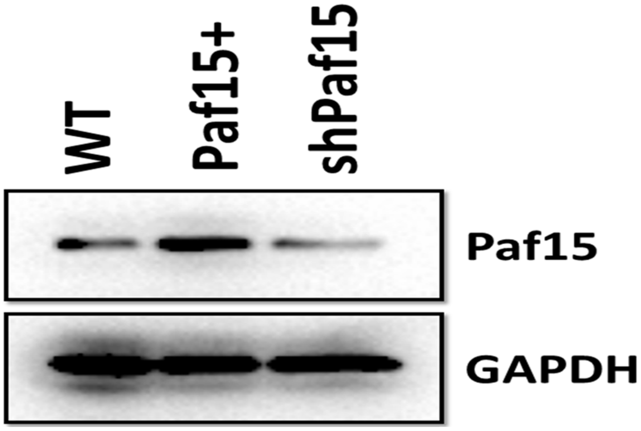

Supplementary Figure S4: Paf15 protein expression after plasmid transfections. RC cells were separately transfected with Paf15+, shPaf15, or empty plasmids for comparison.

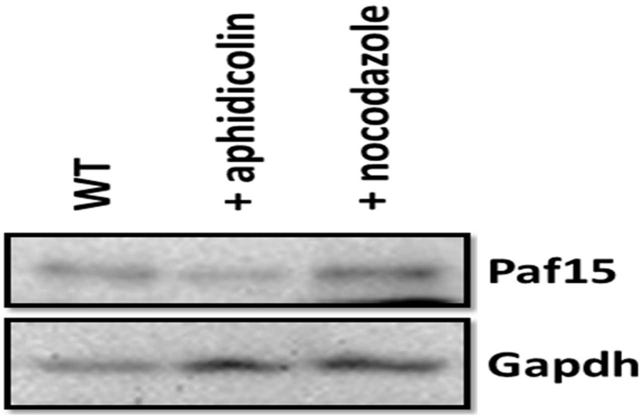

Supplementary Figure S5: Paf15 expression with different cell cycle modifiers. Cell cycle was blocked by aphidicolin in S phase or by nocodazole in G2/M phases, respectively.

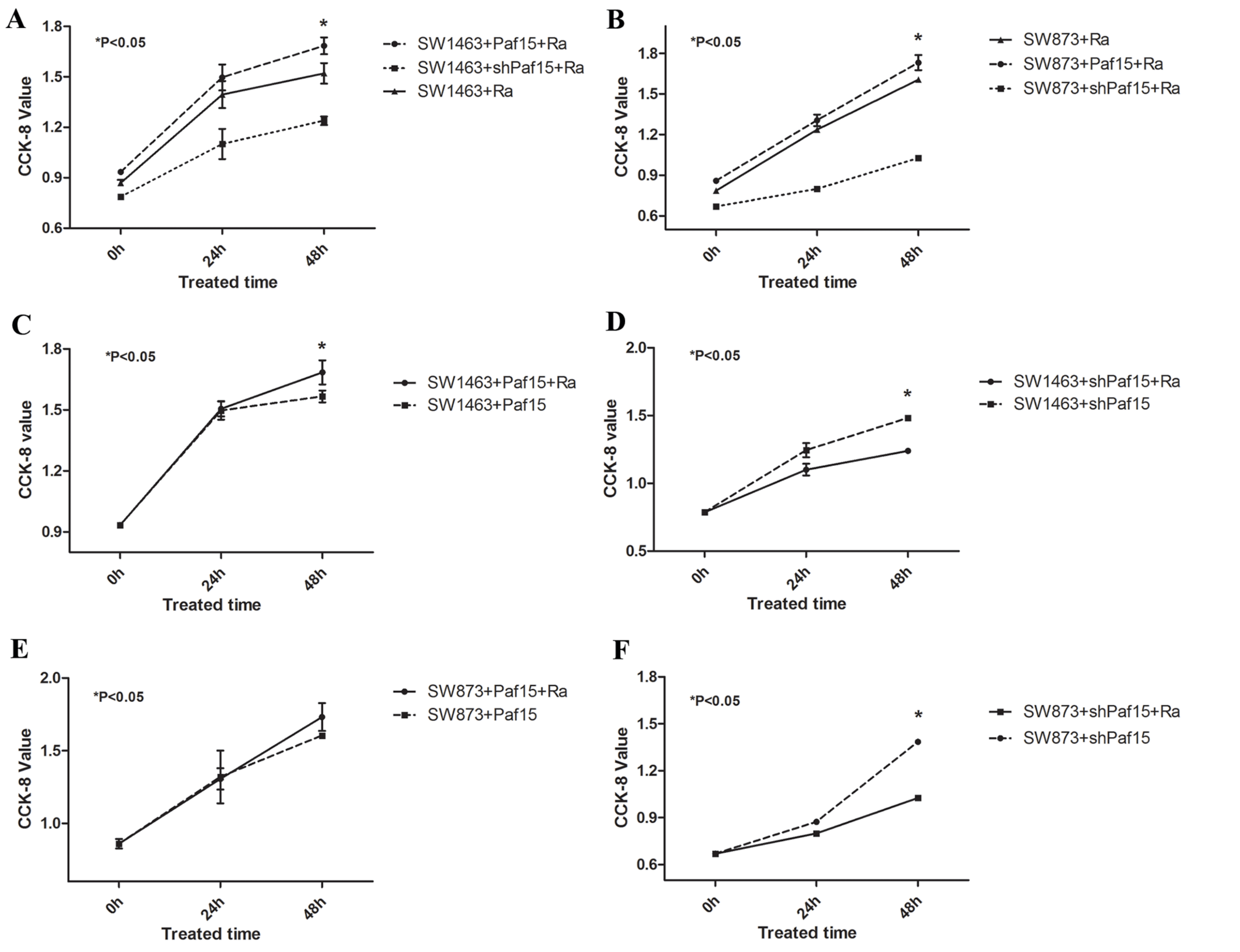

Supplementary Figure S6: CCK-8 assays after irradiation for cells with varying levels of Paf15 expression. A. & **B.** Viability of cells expressing different Paf15 levels after irradiation. **C.** & **E.** Viability of Paf15-high expressing cells under irradiated and control conditions. **D.** & **F.** Viability of Paf15-low expressing cells under irradiated vs. control (unirradiated) conditions.

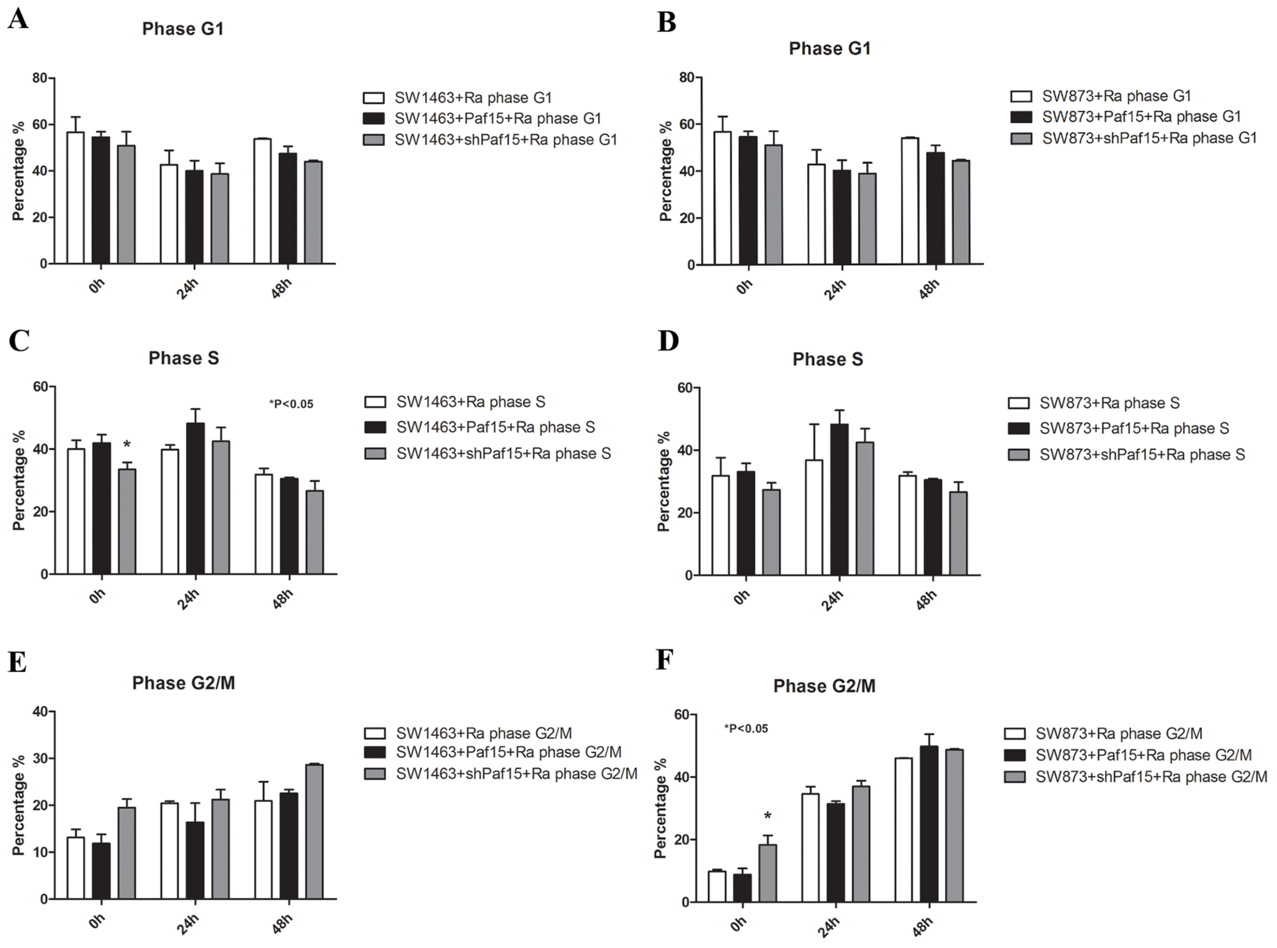

Supplementary Figure S7: Cell cycle distribution at varying Paf15 expression levels. A. & **B.** Percentage of cells in G1phase. **C.** & **D.** Percentage of cells in S phase. **E.** & **F.** Percentage of cells in G2/M phase.

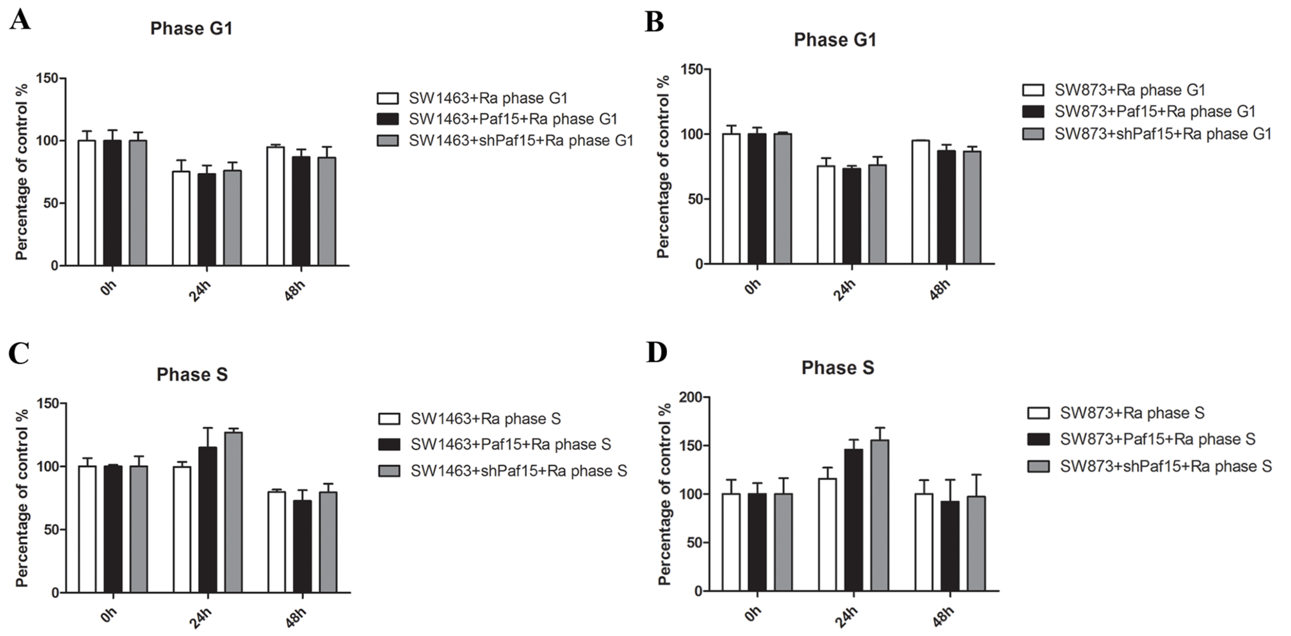

Supplementary Figure S8: Cell cycle after irradiation at varying Paf15 expression levels. A. & **B.** G1 phase cellpercentages of unirradiated controls. **C.** & **D.** S phase cell percentages of unirradiated controls.
